# Dimensionally Specific Capture of Attention: Implications for Saliency Computation

**DOI:** 10.3390/vision2010009

**Published:** 2018-02-17

**Authors:** Katherine E. Burnett, Giovanni d’Avossa, Ayelet Sapir

**Affiliations:** 1 Department of Psychology, Ben-Gurion University, Beersheba 8410501, Israel; 2 School of Psychology, Bangor University, Bangor LL57 2AS, UK

**Keywords:** attention, exogenous cuing, saliency signal, luminance, motion discrimination, color discrimination

## Abstract

Observers automatically orient to a sudden change in the environment. This is demonstrated experimentally using exogenous cues, which prioritize the analysis of subsequent targets appearing nearby. This effect has been attributed to the computation of saliency, obtained by combining features specific signals, which then feed back to drive attention to the salient location. An alternative possibility is that cueing directly effects target-evoked sensory responses in a feed-forward manner. We examined the effects of luminance and equiluminant color cues in a dual task paradigm, which required both a motion and a color discrimination. Equiluminant color cues improved color discrimination more than luminance cues, but luminance cues improved motion discrimination more than equiluminant color cues. This suggests that the effects of exogenous cues are dimensionally specific and may not depend entirely on the computation of a dimension general saliency signal.

## 1. Introduction

Sudden changes in the environment evoke an orienting response, resulting in both greater accuracy and shorter response time to stimuli at the attended location, e.g., [1,2,3,4]. When attention is oriented reflexively to a salient stimulus, such as a new object onset or luminance change, its effects are thought to generalize to all target dimensions [4,5,6,7], even when the orienting stimulus is task-irrelevant and provides no information about the target’s upcoming location [8,9,10,11,12]. An influential theory suggests that local changes in luminance, color, motion etc. are combined within one spatial map, which highlights the position of the most salient aspects of the scene. This map guides a serial search through conspicuous image features in a “winner-take-all” manner [13,14,15].

Whether all visual dimensions are similarly effective in capturing attention, however, remains debated. Theeuwes [16] found that luminance changes were found to evoke covert attentional shifts reliably, but equiluminant color changes did not. Comparing two cue types [17], a color onset and an equiluminant color change, revealed a validity effect following the onset of color cues, but no significant validity effect following a brief equiluminant changes in color. Others studies did report, however, attentional capture by equiluminant color stimuli [17,18,19,20]. More recently, equiluminant color changes lasting 75 ms or longer were found to capture attention [21].

Though equiluminant color cues have been shown to capture attention, luminance changes may have larger effects, i.e., [18,20]. Steinman et al. [20] compared luminance and color cues, and concluded that both cues capture attention, but that luminance cues are more potent and their effects override those of equiluminant color cues. In contrast, Snowden [19] compared reaction times for discriminating the orientation of a luminance target, following partially valid color and luminance cues and found large validity effects for all cues, suggesting that equiluminant and luminance cues were equally effective in capturing attention.

When differences between luminance and color cues were found, they were attributed to differences between magno- and parvo-cellular pathways. Previous research suggests that attentional effects may be greater in the magnocellular stream, where luminance and motion are preferentially processed, over the parvocellular stream, which processes color and shape [20,22,23]. For example, Theeuwes [16] has suggested that the magnocellular pathway transmits signals quickly, leading to prompt reorienting of attention, whereas the parvocellular pathway is sluggish and is therefore not as effective.

While some theoretical models of bottom-up attention suggest that abrupt onset in an otherwise uniform field should produce cueing effects that are not graded [13,15], empirical data suggests otherwise. For example, Burnett, d’Avossa and Sapir [24] found that the size of an exogenous cue can affect the perceptual appraisal of a target. In a dual-task paradigm, participants discriminated the direction of coherent motion of one of four large random dot kinomatograms (RDKs) and localized a small colored probe. The targets were preceded by either a large or a small non-informative exogenous frame cue. Following both large and small cues, discrimination of motion was better at cued than uncued locations. However, localization of the red dot probe was better at cued than uncued locations following the small, but not the large cue. These results suggest that the size of the cue alters the attentional field, and that attentional effects depend on both the size of the exogenous cue and the spatial properties of the task.

There is also evidence to suggest that other properties of exogenous cues, such as luminance contrast, can affect attention [20,25]. Fuller et al. [25] manipulated the contrast of a peripheral cue placed above the target location, and measured the perceived contrast and motion speed of Gabor stimuli in cued and uncued locations. As cue contrast increased, the attentional effects increased, even when the cue contrast was well above detection thresholds. These findings suggest that cueing effects are graded.

Exogenous cueing has also been investigated under conditions in which the task set was manipulated. It has been shown that attentional capture is more likely following cues whose defining feature is the same as the target’s [18,26,27,28,29,30], prompting the hypothesis that attentional capture is contingent on the task set. For example, Folk et al. [28] presented cues followed by targets, where both cues and targets consisted of either an abrupt color or an abrupt luminance change. A validity effect was present when the cue and target were defined by a change along the same visual dimension, whether luminance or color, but not otherwise. Furthermore, it has been shown that participants can maintain multiple task sets across different spatial locations [31,32], suggesting flexibility not traditionally associated with exogenous attention. 

The effects of cue size [24], contrast and luminance [20,25] indicate the contribution of cue properties to exogenous attentional effects. This suggests that exogenous attention is modulated bottom-up, by low-level features of the cue [24]. Moreover, the influence of task set [18,26,27,28,29] indicates that top-down signals also modulate exogenous attention. 

In the present study, we investigate how the similarity of cue and target affects the validity effect, under conditions where cue dimensions are always task relevant. We reasoned that, when cue dimensions are task relevant, one should be able to determine how the degree of similarity between the cue and target dimensions affect attentional capture in a purely bottom-up fashion. A luminance and an equiluminant color change were used as cues in separate blocks [22,23,33], followed by a dual-task in which the observer had to discriminate two target dimensions: motion direction and color. The motion and color targets appeared either in the same or in separate apertures. As the tasks required the analysis of both magnocellular and parvocellular information, the contingent attentional capture hypothesis would predict that both luminance and equiluminant color changes should capture attention [30]. Alternatively, if the visual dimension of the cue biases processing preferentially within dimensionally specific channels, then its effect should depend on the congruency between the visual dimensions of the cue and target. Specifically, the motion task should show a greater validity effect following the luminance than the color cue, and the color task should show a greater validity effect following the color than the luminance cue.

## 2. Materials and Methods

### 2.1. Participants

Thirty-four undergraduate participants were recruited (22 female, mean age 23.47, standard disivation (SD) 6.18). Nineteen were selected via the online participant panel and received course credits for their time, and 15 replied to an advert on the student intranet at Bangor University and were paid for their time. The number of participants was determined by a bootstrapped power analysis, based on the assumption that the validity effect, when cue and target were congruent, was between 5% and 6%, and would half when incongruent cues preceded the target. The power analysis indicated that the number of participants should be between 25 and 37. All procedures were approved by the Ethics Board in the School of Psychology, Bangor University (approval number 1321-A905).

### 2.2. Stimuli and Apparatus

Stimuli were created using Matlab 7.6 (The MathWorks, Inc., Natick, MA, USA) with Psychophysics toolbox extensions [34,35] and generated by an Apple Mac Pro 1.1 computer (Cupertino, CA, USA). Stimuli were displayed on a LaCie Electron 22blue IV (Lacie, Paris, France), 22 inch CRT (Cathode-Ray Tube) screen, with a refresh rate of 60 Hz. The monitor was calibrated and gamma corrected. Head position was restrained by a chin rest at a distance of 70 cm from the monitor. The stimuli were shown against a black background, and participants were in a dark environment.

RDKs of 10° diameter were presented, one in each quadrant of the screen, at an eccentricity of 14° from the central fixation point. Each RDK contained 100 orange dots, 0.2° in diameter. The dots’ initial position was determined by sampling a pseudorandom uniform distribution over the circular window of each RDK. The dots were displaced at a speed of 15°/s in a random direction and had a lifetime of three frames. This process created dynamic noise. In each trial, a 200 ms period of coherent motion was inserted in one of the RDKs. During this time, a proportion of the dots were displaced either up, down, left or right. The proportion of coherent dots was set using a threshold, staircase procedure for each participant at the beginning of the session.

In one of the four RDKs, the dots changed to either red or green for 100 ms. An equiluminance procedure was run to find the subjective ratio for green compared to red for each participant. Following this, in the training and the experiment, red was set at [255 0 0] and green set as the equiluminant [0 (255 × ratio from the equiluminance program) 0]. Following piloting, these colors were then mixed slightly (80% of the original color with 20% of the opposing color) to increase difficulty of the discrimination task. All of the rest of the dots, other than the red or green color discrimination dots, were created by mixing the red and green RGB values together to create an orange shade that was equal in luminance to both the red and the green.

The number of coherently moving and color changing dots was determined using a 2 down, 1 up, staircase procedure. In this session no cues were presented and participants received feedback. The threshold at which participants were 60% correct at each discrimination task was selected for use in the main experiment. Color change dots were selected from the RDK dots independently of the coherent motion dots, so that if both tasks appeared at the same location, color change dots included both translating dots and randomly moving dots. Similarly, coherently moving dots contained both orange and color change dots.

**Figure 1:**
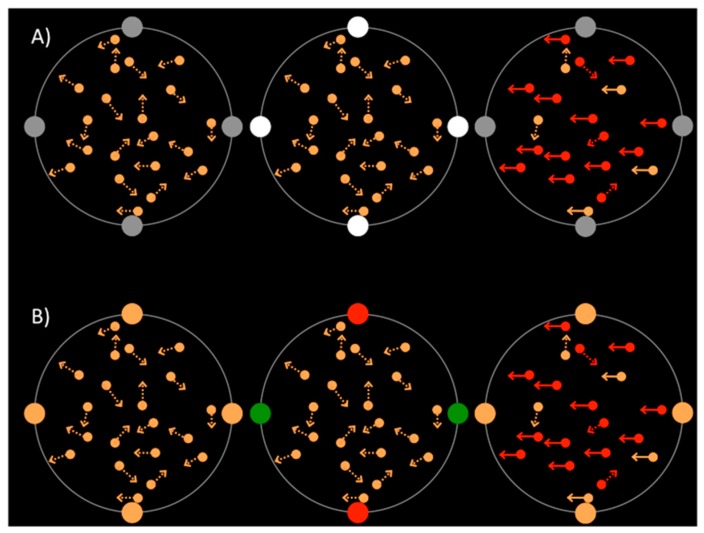
Schematic illustrations of (**A**) luminance and (**B**) color cues. Four dots were present throughout the trial as on the left and right rows, and changed in luminance or color during the cue period, as shown in the center row.

Thin, grey, annular placeholders, 11° in diameter, surrounded each RDK, on which four dots of 2° in diameter were placed as illustrated in Figure 1. In the luminance cue trials, the cue dots were grey, then increased in luminance for 80 ms before returning to grey. In the color cue trials, the cue dots were orange, then two changed to red and two changed to green for 80 ms before returning to orange.

### 2.3. Design

Trials were blocked by cue condition, so that each participant completed 384 trials of the luminance cue and 384 trials of the color cue. The task was identical in both cue conditions; participants reported the direction of coherent motion and the color of the color change dots on every trial.

### 2.4. Procedure

Participants were tested in a 2-h session, which included training on both tasks and two experimental blocks amounting to 768 trials. Participants first completed an equiluminance procedure, in which a freely-available script was modified, based on the minimum motion luminance measurement procedure [36]. Briefly, the stimulus consisted of a complex, annular grating comprising two counter-flickering, superimposed visual patterns. The first component contained two spatially offset cosine waves, defined respectively over red and green phosphors only, such that, where one phosphor peaked, the other troughed. The amplitude of the luminance profile of one phosphor was kept constant, while the second was varied across trials. The second component was a grey level, sinewave grating in quadrature phase with the color gratings, across both space and time. When the two components were superimposed, the annulus appeared to rotate either clockwise or anticlockwise unless the luminance of the two phosphors was matched. This rotatory motion was driven by the luminance difference in the two phosphors; when the luminance of the red was larger than the green, the annulus appeared to rotate clockwise, whereas, if green was larger than red, the annulus appeared to rotate anticlockwise. Participants reported the direction of motion as clockwise or anticlockwise, and responses were used to compute a cumulative probability function. The ratio of red to green luminance at which participants were equally likely to report clockwise and anticlockwise motion was the point of equiluminance. In the first block of trials, well-spaced luminance ratios were used. Subsequent blocks used decreasing increments of luminance values to focus in on the equiluminance point.

Participants then completed 200 trials on the motion discrimination staircase procedure, and 200 trials on the color discrimination procedure. These trials were used to determine the coherence of the translating motion and color change dots in the experiment.

**Figure 2:**
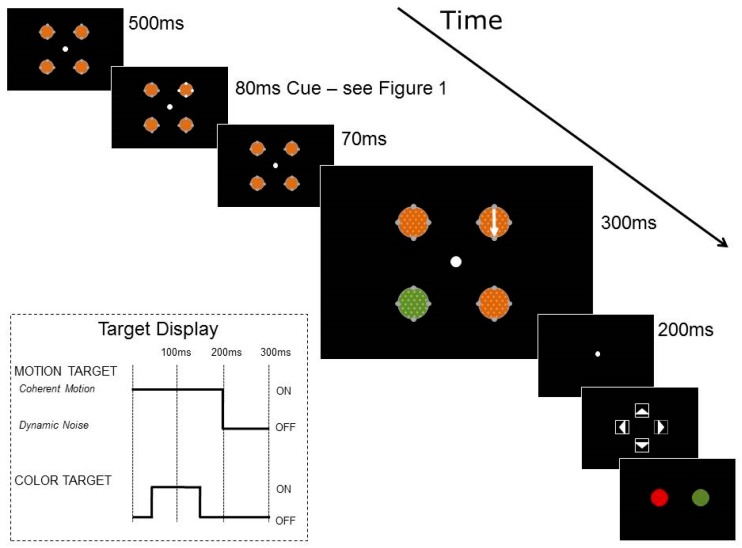
Trial structure for cueing experiment with motion discrimination and color discrimination, cues are shown in more detail in Figure 1. Trial shown is a luminance cue trial, in which motion appears at the cued location and color appears at an uncued location.

Trials were organized into two blocks, based on cue condition: luminance or color. The trial structure is shown in Figure 2. Each trial began with a fixation point in the center of the screen and participants were aware that moving their eyes would make the tasks more difficult because of the speed of the trial. RDKs were visible for 950 ms, starting at the beginning of each trial and surrounded by placeholders and cue dots. After 500 ms of fixation, the cue dots changed in luminance or color for 80 ms, as shown in Figure 1. The two targets then appeared in the RDKs, with 200 ms of coherent motion 150 ms after cue onset, and 100 ms of color change 200 ms after cue onset. The coherent motion and the color change could appear in the same or separate RDKs. At the end of each trial, participants reported first the direction of coherent motion and then the color change.

## 3. Results

Two participants were excluded because they showed no validity effect, following a congruent cue, on both the motion and color tasks (e.g., discriminating motion following a luminance cue), suggesting that the cue had not been effective in attracting their attention. As the main aim of the experiment was to test whether the difference between cue and target dimensions diminishes the validity effect, the logical prerequisite was the presence of a validity effect in the congruent condition. An additional participant was excluded because his discrimination performance was at chance in the color task. Group average accuracies in the motion and color tasks are shown in Table 1.

**Figure 3:**
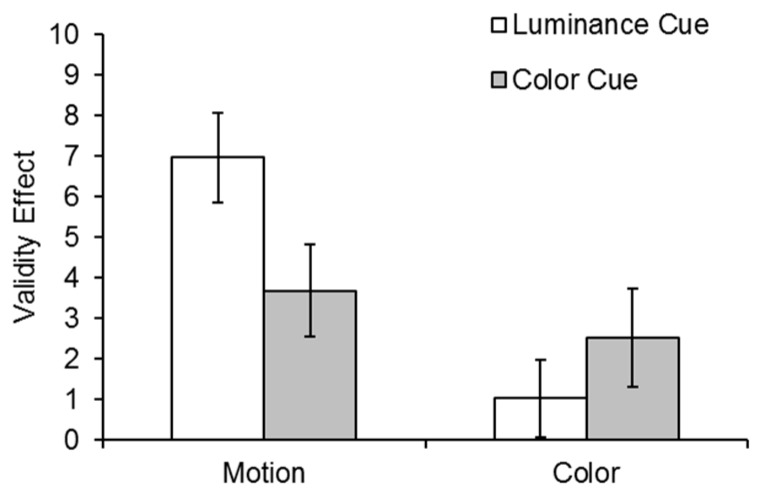
Validity effects in the motion and color discrimination tasks were calculated by subtracting the percent correct on uncued trials from percent correct on cued trials. White bars represent the validity effect following a luminance cue, and grey bars represent the validity effect following a color cue. Error bars represent standard error of the means.

The validity effects for the motion and color tasks in both cueing conditions are shown in Figure 3. Data were analyzed in a 2 (cue condition: luminance, color) × 2 (task: motion, color) × 2 (cue validity: cued, uncued) repeated measures ANOVA. There was a main effect of validity, *F*(1,30) = 32.48, *p* < 0.001, η_p_2 = 0.52, and task, *F*(1,30) = 9.95, *p* = 0.004, η_p_2 = 0.25. The effect of task reflects the greater accuracy in the motion then color task. The interaction between task and validity was also significant, *F*(1,30) = 10.36, *p* = 0.003, η_p_2 = 0.26, reflecting the larger attentional effects for the motion task than the color task. Importantly, there was a 3-way interaction of cue condition, task and validity, *F*(1,30) = 6.37, *p* = 0.017, η_p_2 = 0.175, indicating that the validity effects for the motion and color tasks were different in the two cueing conditions. All remaining comparisons were non-significant. We also performed the same statistical analysis including all 34 participants. All main effects and two-way interactions that were significant in the analysis above were also significant after including all participants. Similarly, all main effects and two-way interactions that were not significant, remained non-significant. In the inclusive analysis, the three-way interaction was marginally significant (*F*(1,33) = 3.95, *p* = 0.055, η_p_2 = 0.107).

In order to examine the 3-way interaction, two 2 (cue condition: luminance, color) × 2 (validity: cued, uncued) repeated measures ANOVAs were conducted separately for the two tasks (motion and color discrimination). In the motion task, there was a significant validity effect *F*(1,30) = 43.14, *p* < 0.001, η_p_2 = 0.59, and a Cue Condition × Validity interaction, *F*(1,30) = 4.66, *p* = 0.039, η_p_2 = 0.13, indicating that the motion validity effect was larger following a luminance than a color cue. However, in the color task, while the validity effect was significant *F*(1,30) = 4.34, *p* = 0.046, η_p_2 = 0.13, the Cue Condition × Validity interaction was not significant, *F*(1,30) = 1.21, *p* = 0.28, η_p_2 = 0.04, suggesting equal validity effects (see Figure 3).

Planned *t*-tests confirmed significant validity effects for the motion task following both a luminance cue, t(30) = 6.36, *p* < 0.001, and a color cue, t(30) = 3.25, *p* < 0.003. However, there was a significant validity effect for the color task following a color cue, t(30) = 2.1, *p* = 0.044, but not a luminance cue, t(30) = 1.07, *p* = 0.29.

We performed a post-hoc power analysis, using a bootstrap procedure. We assumed that the average performance (see Table 1) characterized performance of each of 31 participants, whose measured accuracies reflected the outcome of random sampling with replacement (over 96 valid and 288 invalid trials). For each bootstrapped replication, the *F* value was obtained by computing the ratio of the mean squared interaction effect and the within variance. One hundred thousand replications were generated. The proportion of bootstrapped *F* values greater than the critical *F* value (*F*(1,30) = 4.167) defined the power of our experimental design. The estimated power is 0.72.

## 4. Discussion

We examined the effects of equiluminant and luminance exogenous cues on motion and color discrimination and found an interaction between cue and target dimensions. Following a luminance cue, there was a validity effect for the motion task but not the color task, while following a color cue, both tasks showed a validity effect. This suggests that the similarity between the dimensions of the cue and the target modulate the validity effect. In addition, the validity effects were larger in the motion than in the color discrimination task, adding to evidence of a motion and color asymmetry in attention networks.

### 4.1. Dimension-Specific Cueing Effects

The key finding in this experiment is that low-level visual properties of exogenous cues influence attentional effects. The validity effects in the motion and the color tasks were modulated by whether the cue was a luminance or an equiluminant color change, suggesting that cues which engage the same visual channel as the target are more effective in enhancing target processing at the cued location. 

These effects cannot be accounted for by a dimension general saliency map. That is, if the allocation of attention depends solely on a saliency signal computed by integrating over features and dimensions, one should not expect an interaction between the cue and the target dimensions, contrary to our results. The idea of a saliency map guiding the allocation of exogenous attention could be consistent with luminance and equiluminant cues not being equally effective, as well as the color and motion tasks being differentially susceptible to cuing effects. However, the saliency map model [13,15] would predict that if a cue is less effective in driving attention to a motion stimulus, it should also be less effective in driving attention to a color stimulus, and vice versa (but see the distinction between salience maps and priority maps [37,38]). This prediction is inconsistent with the finding that, while luminance cue was very effective in driving attention to the location of the motion stimulus, it was not effective in driving attention to the location of the color stimulus, whereas the equiluminant color cue was effective in driving attention to the location of both stimuli.

A possible explanation of our finding is that attentional effects simply reflect a feed-forward activation of dimension specific sensory representations. That is, cueing effects may arise as consequence of spatial and temporal co-occurrence of cue and target signals within the same neural structures. This proposal could account for the fact that cueing effects are, to some extent, dimensionally specific, since when the cue and the target do not share the same visual dimension, the likelihood of integrating the two signals within the same structure is diminished, thus resulting in diminished cueing effect.

Our results are compatible with previous findings that attentional effects are modulated by the properties of the exogenous cues, e.g., [20,25]. However, in most studies that examined cue-target similarity, the effects were attributed to attentional control settings [18,26,27,28,29,30]. That is, cues that share the target dimension are more likely to capture attention than cues defined by task-irrelevant dimension. In our study, both visual dimensions were task relevant and, therefore, any difference in cueing effect must reflect a bottom-up effect. We found reduced validity effects in the color discrimination task following the luminance cue compared to the equiluminant cue. This suggests that the effects of the luminance cue did not generalize across target dimensions. This finding is in contrast with other studies, which have reported that a luminance cue can affect the perceptual appraisal of an equiluminant target, e.g., [18,20]. For instance, Lambert et al. [18] found that a luminance cue speeded the detection of an equiluminant target at the cued location compared to targets at the uncued location. However, as target equiluminance was established on physical rather than perceptual grounds, i.e., the authors did not use subjectively equiluminant stimuli, it is possible that, in those studies, the target could be detected using both luminance and chromatic contrasts. 

### 4.2. Motion vs. Color Asymmetry

Larger validity effects were found in the motion than the color task, following both cue types. This is consistent with previous behavioral [39] and imaging findings [40,41,42,43]. For example, participants show greater and more widespread activations when attending to the motion rather than color of the same stimulus [41,42]. Additionally, orientation selective neural responses are increased to targets at the attended location regardless of whether the observers discriminate the target’s orientation or color, whereas color selective neural responses are increased only when observers discriminate the target’s color but not its orientation [43]. This suggests that attentional effects, whether exogenously or endogenously driven, may be greater within the magnocellular than parvocellular stream. On the other hand, our results may be less consistent with the proposal that equiluminant exogenous cues are less effective than luminance cues, as this was the case only in the motion discrimination task. Therefore, one may conclude that saliency may not differ greatly between luminance and equiluminant stimuli, but that attention more readily affects target processing in the magno than parvocellular pathway.

A potential confound limits the interpretation of our findings. Discrimination accuracy differed between motion and color tasks. It is possible that this difference may account for the greater susceptibility to cueing effect of the motion than the color task. We think that this is an unlikely account of our findings as the properties of the psychometric curve should predict that the task performed less accurately would benefit more from cueing than the more accurate task, e.g., [44]. Nevertheless, if one were to compare cueing effects across, rather than within tasks, it would be crucial to ensure that cueing effects are assessed across a broad range of difficulties, by varying the signal-to-noise ratio of the targets. This manipulation would provide a stronger measure of cueing efficacy and shed light on the mechanisms underlying cueing effects [45].

## 5. Conclusions

In conclusion, we showed that low-level dimension similarity between a cue and subsequent targets modulates attentional effects. This contradicts the view of exogenous attention as a unitary process [46]. We propose that dimensional specific cueing effects reflect bottom-up interaction between the cue and the target.

## Figures and Tables

**Table 1:** Mean accuracy (%) for motion discrimination and color discrimination tasks on cued and uncued trials.

	Motion		Color	
	*M* (SD)	95% CI ^1^	*M* (SD)	95% CI ^1^
Luminance cue				
Cued	78.29 (10.84)	[74.3, 82.3]	71.48 (11.22)	[67.4, 75.6]
Uncued	71.32 (10.19)	[67.6, 75.1]	70.45 (8.67)	[67.3, 73.6]
Color cue				
Cued	76.58 (10.62)	[72.7, 80.5]	69.9 (9.81)	[66.3, 73.5]
Uncued	72.9 (9.39)	[69.5, 76.3]	67.3 (7.55)	[64.6, 70.2]

^1^ CI = confidence interval.
